# Milling of an Al/CFRP Sandwich Construction with Non-Coated and TiAlN-Coated Tools

**DOI:** 10.3390/ma13173763

**Published:** 2020-08-26

**Authors:** Elżbieta Doluk, Anna Rudawska, Józef Kuczmaszewski, Paweł Pieśko

**Affiliations:** Department of Mechanical Engineering, Lublin University of Technology, 20-388 Lublin, Poland; a.rudawska@pollub.pl (A.R.); j.kuczmaszewski@pollub.pl (J.K.); p.piesko@pollub.pl (P.P.)

**Keywords:** Al/CFRP, milling, cutting parameters, TiAlN coating

## Abstract

In this study, the effect of cutting parameters on the quality of an Al/CFRP sandwich structure (aluminium alloy–carbon fibre reinforced polymer) after milling with uncoated and TiAlN-coated tools was examined. The results of the cutting force were also investigated. The research was conducted in a VMC 800 HS vertical machining centre with a variable cutting speed and feed. The milling process was carried out using a non-coated, two-blade carbide milling cutter with a 35° helix angle and an analogous tool with a TiAlN coating. The surface quality was characterised in terms of the height deviation, which is one of the shape deviations after machining hybrid materials. The research showed that the maximum (77.60 µm) and minimum (1.78 µm) values of the height deviations were obtained using the tool with a TiAlN coating. It was found that the tested factors had significant effects on the height deviation, where the feed had the greatest influence and the cutting speed had the lowest influence on the surface quality. The tested factors were not statistically significant in terms of the cutting force.

## 1. Introduction

Sandwich structures have been used in many industries, in particular, in the marine, aviation and automotive sectors. This is due to their advantages, including low weight, high mechanical properties and flexural stiffness [1]. Because of this, sandwich materials have become a common alternative to solid construction. An example of a sandwich that is a promising solution for many areas is a hybrid structure consisting of a metal and polymer composite. The main purpose of machining this type of material is that the workpiece meets dimensional requirements. The sandwich structures are usually formed into the desired shape and are processed with the same methods as CFRP (carbon fibre reinforced polymer), i.e., milling, drilling and grinding. The sandwich structures are difficult to process. This is due to the fact that it consists of different materials, which means that the machining is characterised by different material removal mechanisms [2]. Problems already arise at the cutting stage of the composite material because the reinforcement and matrix have different properties: the fibers are strong and brittle, while the matrix is weak and ductile [3]. As a result, the cutting forces are not continuous. This causes defects on the machined surface and intensive tool wear [4].

In [5,6], it was pointed out that one of the main factors affecting the machinability and tool wear of CFRP is the fiber orientation. Milling of the Al/CFRP sandwich structures (aluminium alloy–carbon fibre reinforced polymer) requires the use of appropriate cutting parameters to reduce the labor and operating costs. Santhanakrishna et al. [7] studied the influence of the cutting speed, feed rate and cutting width on the cutting forces and the surface roughness after machining CFRP. The cutting force value can be also used to evaluate the machinability of Al/CFRP stacks. Rahman [8] noted that the cutting force and surface quality of CFRP depend on the cutting parameters. Ramulu et al. [9] noted that the feed rate has the greatest impact on the surface quality and the selection of the optimal cutting parameters improves the quality of the workpiece. In [10], it was noted that lower cutting forces were obtained for higher cutting speeds and lower feed rates. Uhlmann et al. [11] studied the effect of the cutting force on the chip formation mechanism during the milling of CFRP. The results showed that a higher cutting speed leads to a reduction in the processing forces. This results in a prolongation of the tool life or the possibility of using higher feed rates.

Bivolaru et al. [12] stated that a change in the cutting speed without changing other cutting conditions results in different surface roughness parameters. They also observed that an increase in the feed rate increases the cutting forces. The tool geometry also has a great influence on the machinability of the hybrid sandwich structures. Manufacturers offer newer solutions, such as compression mills (dedicated especially for workpieces with a strong delamination tendency), multi-bladed milling cutters, tools with a new cutting edge geometry (helix angle), pineapple-like shape tools or tools with additional notches on the teeth [13].

Surface geometry after machining is the result of several factors. One of them is the machine tool kinematics, which is mainly influenced by the tool wear machine vibrations and inhomogeneity of the workpiece [14]. The processing of the Al/CFRP could result in unsatisfactory quality and dimensional accuracy of the workpiece compared to the processing of single materials. The reasons are the different properties of materials, different machinability, different chip formation mechanisms, different machining stability and interactions between the layers [15]. The machining of machine parts involves the necessity to fit within certain tolerance ranges. Dimension, shape and position deviations beyond these ranges can cause machine components to malfunction and make their assembly difficult or impossible. The selection of optimal cutting conditions allows for minimising the occurrence of these deviations. There has been a lot of research on the surface quality of the Al/CFRP stacks after machining. Most of the researchers focused on studying the surface roughness [16,17] or delamination [18]. Few studies have described the shape deviation and the reasons for their formation. Denkena et al. [15] mentioned three shape deviations that can be used to assess the surface quality of sandwich constructions: material height deviation, transition deviation and surface roughness deviation. Moreover, a visual inspection is also used to spot defects, such as delamination, debonding, fiber pull-out or spalling. In [19], they focused on the shape deviation (including the height deviation) after parallel machining. However, they did not investigate the effects of cutting conditions on the values of deviations. The Al/CFRP stacks have been widely used in aviation as aerospace structural elements, such as aircraft wings and tail-plane components. The sandwich structures used in aviation are usually coated with protective, decorative or technical coatings. In surface engineering, a height deviation of even a few microns can make a significant difference. A high value of the height deviation may result in an insufficient adhesion of a coating and reduce its durability. Furthermore, a height deviation can reduce the visual effect of decorative coatings. It is also important in terms of obtaining the correct fit of the elements. These types of materials are at times bonded in large-sized structures, where height deviations may affect the strength of the adhesive joints. The quality after milling is defined by the surface finish, surface integrity and homogeneity of the defect, where a metal layer has a different quality than a CFRP layer [14]. One way to achieve a high-quality surface after the milling process is to reduce the cutting forces. This allows for minimizing the tearing of the fibers, but on the other hand, causes a decrease in the processing efficiency (reduction of the feed rate). Another way is to adjust the cutting parameters to the currently processed material. Unfortunately, the use of variable cutting parameters does not work in all machining conditions. The next solution is to use coated tools. A properly selected tool coating increases the durability of the cutting blade, which results in prolongation of the tool’s life and ensures the required dimensional accuracy and affects the cost of machining [20,21]. In [22], the tool life during drilling of the CFRP/Ti stacks was examined, where the authors compared the tool wear for CFRP/Ti with the reference samples (CFRP and Ti). It was noted that the tool wear from the metal was nine times smaller compared to the wear from CFRP. They also noted that after the CFRP machining, there was a different type of tool wear than after the machining of Ti.

Hosokawa [23] presented the results of milling CFRP with diamond-coated tools that had different helix angles. They showed that the occurrence of the defects depends on the cutting force and the tool wear depends on the fiber orientation. On the other hand, the presence of a tool coating is associated with an increase in the layer thickness and edge rounding. Teti [4] recommended that the edge serration and nose radius be as small as possible. Janardhan [24] also showed that the machined quality is affected by the tool nose radius. It should be considered whether the presence of the coating will adversely affect the surface quality after machining.

Despite the increasing use of Al/CFRP structures, many issues have not yet been thoroughly investigated. One of them is the influence of the cutting speed and feed on the height deviation after the milling of an Al/CFRP stack with non-coated and TiAlN-coated tools. Therefore, an attempt was made to compare the effects of milling with coated and uncoated tools using variable cutting parameters.

## 2. Materials and Methods

The subject of this study was a hybrid sandwich structure consisting of two materials: 2024 aluminium alloy and carbon fibre reinforced polymer (CFRP). The materials were selected because of their use in the aviation industry.

The CM-Preg TI02 20/1000 CP00690 epoxy tissue prepreg (trade name), manufactured by the c-m-p gmbh company (Heinsberg, Germany), was used in the experiment. The prepreg areal weight was 200 g/m^2^. The matrix was a thermoplastic modified epoxy resin and the reinforcement was unidirectional, high-strength carbon fibers. The volume fraction of the reinforcement was 60%. The composite was fabricated using an autoclave process (Scholz, Coesfeld, Germany) for 60 min at 130 °C under a 0.4 MPa pressure. The manufacturer determined the following mechanical properties of the CFRP: modulus of elasticity 135 GPa, tensile strength 1900 MPa, flexural strength 2050 MPa and flexural modulus 130 GPa [25,26].

The materials were bonded using Scotch-Weld-9323 B/A adhesive (3M, St. Paul, MN, USA) in a weight ratio of 100:27. The specimens were a plate shape with dimensions of 60 mm × 120 mm × 6 mm (width, length and thickness, respectively). The thickness of the adhesive layer was 0.1 ± 0.02 mm.

**Figure 1:**
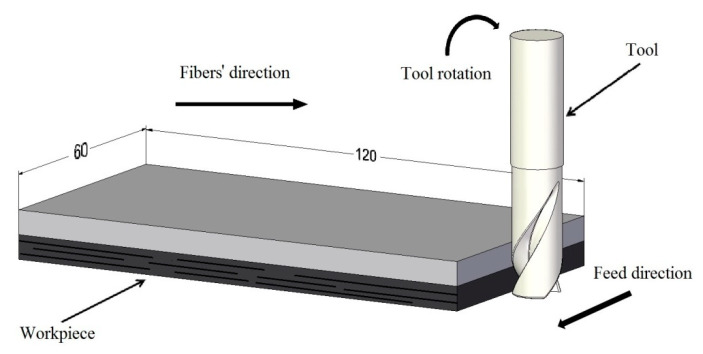
Experimental setup.

The experiment consisted of peripheral down-milling of the short side of the specimen (Figure 1). The process was performed in a VMC 800 HS vertical machining centre (AVIA, Warsaw, Poland). A two-blade carbide milling cutter with a 35° helix angle and TiAlN coating and an analogous milling cutter without a coating (Garant, Munich, Germany) were used. The material of the tools was micro-grain grade carbide consisting of 90% tungsten carbide and 10% cobalt. The tools had a cutting diameter of 12 mm, cutting length of 26 mm, shank diameter of 12 mm, overall length of 83 mm, rake angle of 16°, corner chamfer of 45° and chamfer length of 120 μm (non-coated tool) and 124 μm (TiAlN-coated tool). The coating thickness was 4 μm. The tool and the type of coating were selected due to the fact that they were not dedicated to a narrow range of materials; they can be used for both aluminium and CFRP machining. In addition, due to the low coefficient of friction, the coating reduced the heat flow at the contact point of the tool with the workpiece, thus reducing the heating of the tool blade. This is especially important during the milling of CFRP. Blocking the heat flow leads to prolongation of the tool’s life. The TiAlN coating, compared to non-coated tools, leads to a reduction in flank wear. Moreover, the high hardness and the abrasion resistance reduce oxidation and chemical wear of the material of the blade. The coating also alters the tool wear pattern. The first stage in the wear of coated tools is the abrasive wear of the flank surface. The next stage is a modification of the cutting edge (wear of the rake surface). In the last stage, due to the adhesive wear, the coating is pulled out [27].

In addition, the helix angle λ_s_ = 35° improves the chip evacuation process [28]. This is also important during CFRP machining.

**Figure 2:**
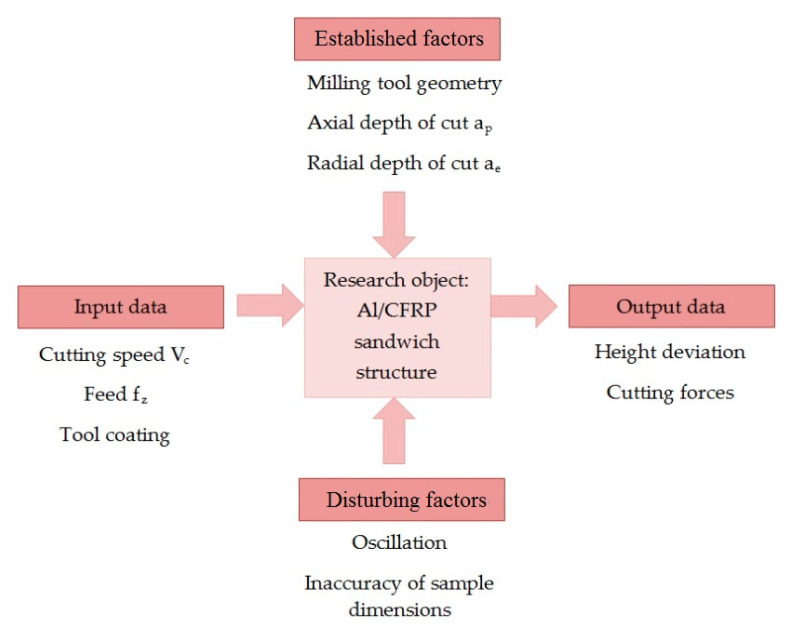
Research plan. CFRP: Carbon fibre reinforced polymer.

The schematic of the experiment is shown in Figure 2. The research was conducted using three input factors (Table 1): two at three levels (V_c_ and f_z_) and one at two levels (coating). The values of the cutting parameters were based on the recommendation of the producer of the tools. The values suitable for the aluminum alloy, CFRP and the intermediate value were selected. The constant parameters were the axial depth of the cut a_p_ = 12 mm and the radial depth of the cut a_e_ = 4 mm. The milling process was repeated two times for each specimen.

Because the cutting forces affect the surface quality [14], their values were recorded during machining. The cutting forces were measured using a Kistler dynamometer, model 9257B (Kistler, Winterthur, Switzerland), mounted in the machine spindle. The device was connected to the specialised software, which allowed for data acquisition. Three cutting force components were recorded: F_x_, F_y_ and F_z_. Because the F_x_ for all samples had the highest values compared to the F_y_ and F_z_, it was subjected to further analysis. The adopted cutting force was the average of three maximum readings.

After the milling process, the influence of the cutting parameters and the presence of the coating on the surface quality of the sandwich construction was examined. The quality was characterised using a type of shape deviation, namely, height deviation. The main reason for this deviation was the different properties of the layers. During the machining, the tool processed two different materials at the same time: aluminum alloy (lower strength, higher density) and CFRP (higher strength, lower density). This created the shape deviations that were different after machining single materials.

During milling of the single material, the tool bent in the feed direction, while milling of the sandwich structure, the tool bent in the feed’s normal direction. This caused a different inclination of the profiles and was caused by the cutting force fluctuation, mainly the passive forces (the cutting force component along the radial direction of the sample), where the higher the passive force, the higher the height deviation. Different material properties result in different passive forces and uneven tool loads. It leads to shape deviations of the workpiece [13].

**Figure 3:**
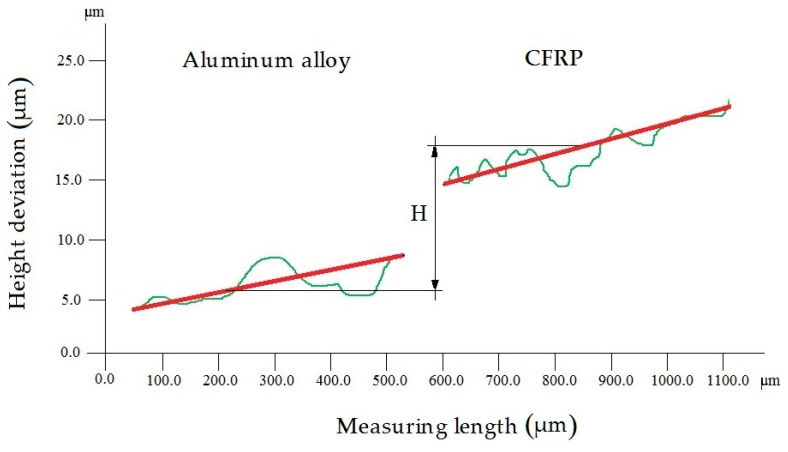
Height deviation of the Al/CFRP sandwich structure.

The height deviation was defined by the distance between the profile of the aluminum alloy and the profile of the CFRP (Figure 3), as follows:

(1) $$H=H_{Al}-H_{CFRP},$$

where:

*H*—height deviation (µm),
*H_Al_*—average profile of the aluminium alloy (µm),
*H_CFRP_*—average profile of the CFRP (µm).

A VHX-500 microscope (Keyence, Osaka, Japan) was used to measure the height deviation of the specimens. The measuring device allowed for recording with a high measurement resolution (0.01 μm). Moreover, it allowed for observations of the geometric structure of the machined surface. The height deviation was captured at a magnification of 500×. The deviation was measured over a measuring length of 0.5 mm × 0.5 mm in the center of the tested sample. The profile was measured at three sample places, and the mean of the measured values was selected for analysis.

## 3. Results

### 3.1. Height Deviation

**Figure 4:**
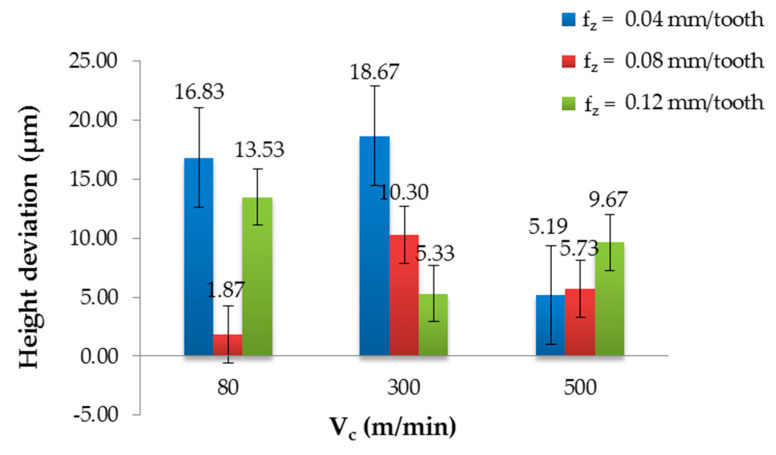
Height deviation in relation to the cutting speed after milling with different feeds and a non-coated tool.

**Figure 5:**
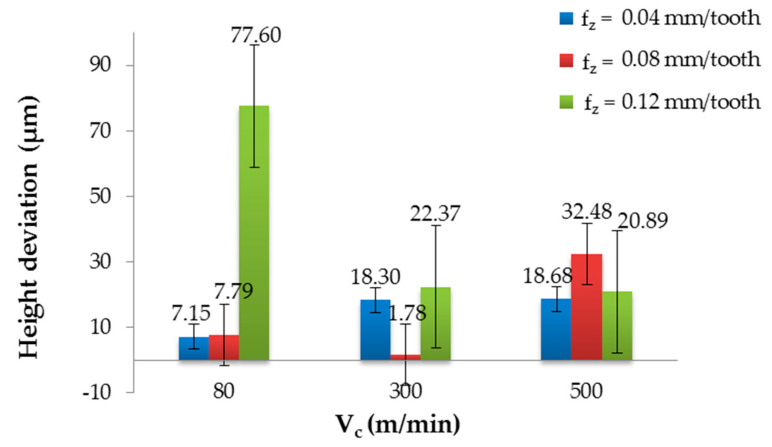
Height deviation in relation to the cutting speed after milling with different feeds and a TiAlN-coated tool.

Figure 4 and Figure 5 show the influence of the cutting parameters on the height deviation for the non-coated and coated tools. The results demonstrated that the cutting conditions and the type of tool affected the quality of the sandwich structure. The non-coated tool allowed for obtaining the maximum value (18.67 µm) using V_c_ = 300 m/min and f_z_ = 0.04 mm/tooth, and the minimum value (1.87 µm) using V_c_ = 80 m/min and f_z_ = 0.08 mm/tooth. The difference between the extremes was nearly 90%. No stabilisation of the height deviation was observed for any of the tested cutting speeds; only V_c_ = 500 m/min allowed for obtaining the most similar values for the tested feed values (Figure 4). It was found that for smaller feed values, the highest height deviation was obtained using V_c_ = 300 m/min. At the highest feed rate, the lowest deviation was obtained at this value of V_c_. On this basis, it was possible to determine the range of recommended cutting parameters due to the accuracy of the machining and machining efficiency (higher feed rate and cutting speed values led to higher machining efficiency). Considering the quality and efficiency parameters, it seemed optimal to use the feed f_z_ = 0.08 mm/tooth and the cutting speed V_c_ = 500 m/min.

It can be seen from Figure 5 that higher values of the height deviation were obtained after processing with the TiAlN-coated tool. The maximum value (77.60 µm) was observed for V_c_ = 80 m/min and f_z_ = 0.12 mm/tooth. The minimum value (1.78 µm) was noted for V_c_ = 300 m/min and f_z_ = 0.08 mm/tooth; the difference between the extremes was about 98%. After examining the results, it can be seen that for f_z_ = 0.04 mm/tooth, the height deviation increased with the increase of the cutting speed, from 7.15 µm to 18.63 µm. The results obtained using f_z_ = 0.08 mm/tooth showed mixed behaviours, where the minimum height deviation at the moderate cutting speed (V_c_ = 300 m/min) was noted. After analysing the results obtained for the f_z_ = 0.12 mm/tooth, a decreasing trend was observed: an increase in the cutting speed caused a decrease in the height deviation. Comparing the results obtained after the milling with the non-coated and coated tools, it was observed that the maximum value obtained for the non-coated tool was nearly 76% lower than the maximum value obtained with the coated tool. The minimum values were similar, where the difference was about 5%.

The influence of the cutting parameters, presence of the tool coating and their interaction on the height deviation was analysed using ANOVA, which is a method for testing the significance of differences between many means of samples from many groups. It can be seen that all factors significantly affected the height deviation within the 95% confidence interval (Table 2). In addition, there were interactions of the factors, where the height deviation response to a change in one factor was not the same for all levels of the second factor. It can be seen that the feed (f_z_) reached the highest F-ratio. On this basis, it can be concluded that f_z_ had the highest effect on the height deviation, followed by the presence of the coating (C), the interaction V_c_ f_z_, the interaction V_c_ × C, the interaction f_z_ × C and the cutting speed (V_c_).

**Figure 6:**
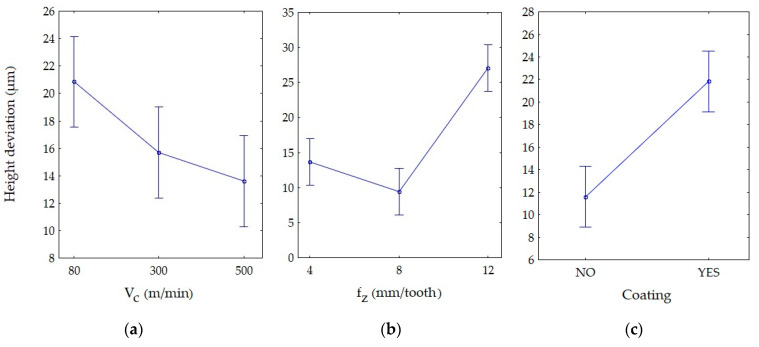
Main effects on the height deviations: (**a**) cutting speed, (**b**) feed and (**c**) presence of the coating.

**Figure 7:**
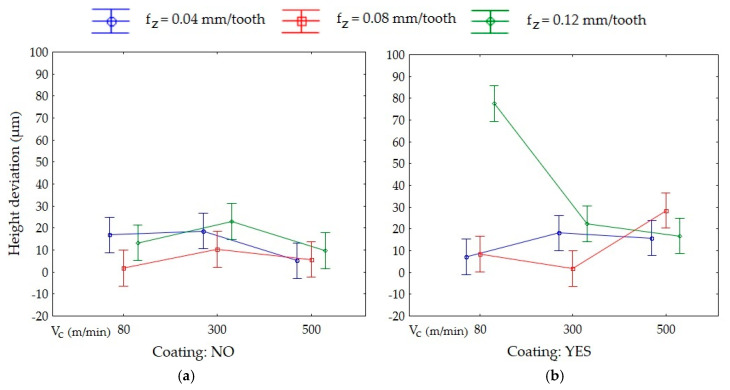
Interaction on height deviation using: (**a**) non-coated tool and (**b**) TiAlN-coated tool.

The differences between the groups were tested using the post hoc Bonferroni’s test. The graphical interpretation of the statistical results are presented in Figure 6 and Figure 7.

Figure 6 displays the effect of the levels of one factor on the shape deviation at the level of another factor. The vertical bars represent the 95% confidence intervals. The modification of the interaction of one factor by another is illustrated by the intersection of the line plots. Parallel lines represent statistically insignificant means, where there are no grounds for rejecting the hypothesis of the means’ equality. If the lines intersect and the confidence bars overlap, the results should be considered statistically significant.

Examining Figure 6, it can be stated that for the cutting speed, the statistically significant difference was between V_c_ = 80 m/min and V_c_ = 500 m/min (Figure 6a). The analysis of the feed (Figure 6b) showed that there were statistically significant differences between f_z_ = 0.12 mm/tooth, f_z_ = 0.04 mm/tooth and f_z_ = 0.08 mm/tooth. The same applied to the results obtained with the non-coated (NO) and TiAlN-coated tool (YES) (Figure 6c).

The vertical bar of one group (V_c_ = 80 m/min, f_z_ = 0.12 mm/tooth, coating: YES) did not overlap with the vertical bars of other groups. Furthermore, there were interactions between the four groups of factors (Figure 7).

### 3.2. Cutting Force

**Figure 8:**
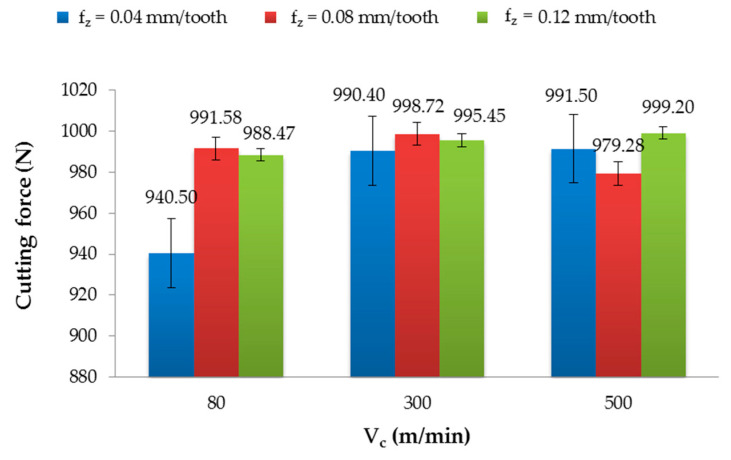
Cutting force in relation to the cutting speed after milling with different feeds and using a non-coated tool.

**Figure 9:**
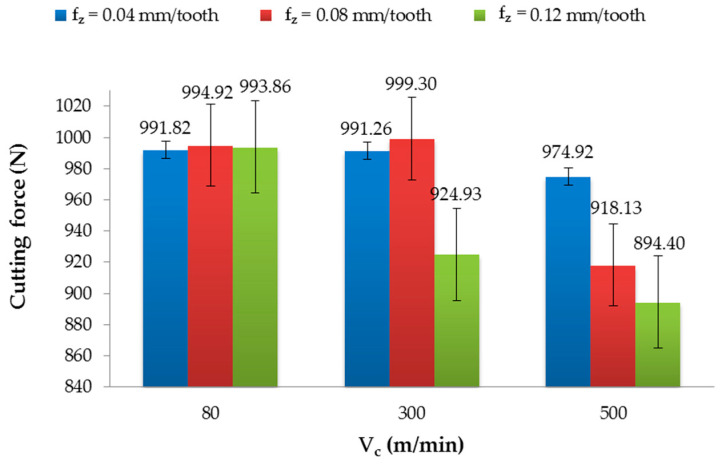
Cutting force in relation to the cutting speed after milling with different feeds and the TiAlN-coated tool.

Figure 8 and Figure 9 present the effect of the tested factors on the cutting force. The maximum value of the cutting force (999.20 N) after milling with the non-coated tool was obtained for V_c_ = 500 m/min and f_z_ = 0.12 mm/tooth, and the minimum value (940.50 N) was obtained for V_c_ = 80 m/min and f_z_ = 0.04 mm/tooth. The difference was about 6%. In this case, for f_z_ = 0.04 mm/tooth and f_z_ = 0.12 mm/tooth, an increase in the cutting speed caused an increase in the cutting forces. For f_z_ = 0.08 mm/tooth, the cutting force increased in the range of V_c_ = 80–300 m/min and then decreased for V_c_ = 500 m/min. (Figure 8).

The parameters that yielded the highest (999.30 N) and lowest (894.40 N) value of the cutting force after milling with the TiAlN-coated tool were as follows (respectively): V_c_ = 300 m/min and f_z_ = 0.08 mm/tooth, and V_c_ = 500 m/min and f_z_ = 0.12 mm/tooth (Figure 9). The difference between the extremes was nearly 10%. Examining the results for f_z_ = 0.04 mm/tooth and f_z_ = 0.08 mm/tooth, it can be observed, as with the non-coated tool, that the cutting forces increased in the range of the cutting speed V_c_ = 80–300 m/min and decreased at V_c_ = 500 m/min. For f_z_ = 0.12 mm/tooth, the increase in the cutting speed caused a decrease in the cutting force.

The results of the cutting force were not normally distributed; therefore, the statistical analysis was carried out based on the Kruskal–Wallis test (H test) with one-way ANOVA on the ranks. The compared factors were taken from populations of the same rank; the differences were not statistically significant (Table 3). It can be concluded that the analysed factors did not affect the cutting force value.

## 4. Discussion

Based on the above results, the influence of the tested factors on the effects of the milling of the Al/CFRP structure was stated. A change in the cutting parameters resulted in a high variation of the height deviation. It was observed that the feed was the most influential factor. This result is in agreement with the literature, where Shahrajabian and Farahnakian [29] reached similar conclusions. They studied the effect of the feed and tool diameter on the surface roughness after machining. They showed that the feed is the most important factor affecting the surface roughness. The cutting speed had the smallest effect on the output data [30,31].

In addition, a significant effect of the presence of the coating was found on the height deviation; for most of the parameter sets, a higher height deviation was obtained after milling with the coated tool. The TiAlN coating increased the nose radius, reduced the sharpness of the cutting edge and led to an increase in the height deviation. This is consistent with some studies [29,32].

Hocheng et al. [5] studied the wear of non-coated and coated end mills in CFRP milling. They showed that for a coated tool, different types of wear were found in comparison to a non-coated tool. They noticed that a satisfactory surface quality was reached with a low feed.

The cutting force results were statistically similar and did not depend on the tested factors. Denkena et al. [19] have noted the opposite conclusion, where the cutting force increases as the feed increases, which causes a height deviation increase. Zitoune et al. [33] studied machining conditions and non-coated tool use during the drilling of CFRP/Al. They observed that the size of the chips was influenced by the feed. They also noticed that the force during the drilling of CFRP with a coated tool was about 15% smaller in comparison to drilling with uncoated drills. They stated a similar conclusion for the aluminum alloy, where the force was 50% smaller using a coated tool in comparison to drilling with a non-coated tool. Wang [34] showed that the cutting force increases with increasing feed rate but depends on the cutting speed. Examining the results of cutting forces, it can be noted that for both types of tools for f_z_ = 0.04 mm/min and for the non-coated tool at f_z_ = 0.12 mm/min, the cutting forces increased with an increase in the cutting speed. These results are consistent with some studies [35,36]. The cutting forces obtained after milling with the TiAlN-coated tool for f_z_ = 0.12 mm/tooth decreased with an increase in the cutting speed. This could probably be related to HSM (high-speed machining). The result obtained at f_z_ = 0.08 mm/tooth for both tools showed a similar tendency, where the cutting forces increased from V_c_ = 80 m/min to V_c_ = 300 m/min, and then decreased at V_c_ = 500 m/min. Examining the height deviation and cutting force results, a similar trend can be seen for half of the tested sets (f_z_ = 0.04 mm/tooth and f_z_ = 0.12 mm/tooth with the TiAlN-coated tool and f_z_ = 0.08 mm/tooth with the non-coated tool). On the other hand, for the other sets, there was no clear relationship between the cutting forces and the height deviation. It is difficult to find the cause of this phenomenon, though it was probably related to the frictional-adhesive mechanism. The physical interpretation of this phenomenon requires in-depth study.

## 5. Conclusions

Based on the obtained results and statistical techniques employed, the following conclusions were drawn:

1. The tested factors (cutting speed, feed and presence of a coating) had a significant effect on the shape deviation.
2. The feed was found to have the highest influence and the cutting speed was found to have the lowest influence on the height deviation.
3. The maximum (77.60 µm) and minimum (1.78 µm) values of the height deviation were observed after milling with the TiAlN-coated tool.
4. It was noticed that for the TiAlN-coated tool at f_z_ = 0.12 mm/tooth, the increase in the cutting speed caused a decrease in the height deviation. For f_z_ = 0.04 mm/tooth, the trend was the opposite.
5. The tested factors did not affect the cutting force; the results were not statistically significant.
6. The maximum (999.30 N) and the minimum (894.40 N) values of the cutting force were achieved using the TiAlN-coated tool.
7. It was found that the cutting forces obtained after milling with the non-coated tool for f_z_ = 0.04 mm/tooth and f_z_ = 0.12 mm/tooth increased with a decrease in the cutting speed.
8. For f_z_ = 0.12 mm/tooth, the cutting force obtained using the TiAlN-coated tool decreased with an increase in the cutting speed.

## Figures and Tables

**Table 1:** Design of experiment.

Factors	Level		
	1	2	3
V_c_: cutting speed (m/min)	80	300	500
f_z_: feed (mm/tooth)	0.04	0.08	0.12
C: coating	NO	YES	

**Table 2:** Two-factor ANOVA for height deviation.

Source	Sum of Squares	df	Mean Square	F-Ratio	*p*-Value
V_c_: cutting speed	500.92	2	250.46	5.19	0.0104
f_z_: feed	3063.14	2	1531.57	31.76	0.0000
C: coating	1412.92	1	1412.92	29.30	0.0000
Interaction V_c_ × f_z_	3538.85	4	884.71	18.35	0.0000
Interaction V_c_ × C	1325.18	2	662.59	13.74	0.0000
Interaction f_z_ × C	1313.04	2	656.52	13.62	0.0000
Total error	1735.77	36	48.22	-	-
Total (corrected)	1288.82	49	-	-	-

**Table 3:** Kruskal–Wallis test results for the cutting force.

Source	df	H	*p*-Value
V_c_: cutting speed	3	1.67	0.4300
f_z_: feed	3	5.23	0.6310
C: coating	2	2.76	0.0967
